# Multimodal medical diagnosis: a mini review of LLM–vision fusion models in low-resource healthcare settings

**DOI:** 10.3389/fdgth.2026.1862236

**Published:** 2026-09-02

**Authors:** Kahakashan Ashraf, Md. Hamid Hosen, Nuzhat Tabassum Farah, Md Kishor Morol, Tze Hui Liew, Dip Nandi, Mashiour Rahman, Abdullah Al Jubair

**Affiliations:** 1 Department of Computer Science and Engineering, Chittagong University of Engineering and Technology, Chattogram, Bangladesh; 2 Department of Computer Science and Engineering, East Delta University, Chattogram, Bangladesh; 3 ELITE Research Lab, LLC, Queens, NY, United States; 4 Faculty of Information Science and Technology, Multimedia University, Bukit Beruang, Melaka, Malaysia; 5 Department of Computer Science and Engineering, American International University-Bangladesh, Dhaka, Bangladesh

**Keywords:** clinical AI deployment, LLM-vision fusion, low-resource healthcare, medical imaging, multimodal medical diagnosis, transformer architectures, vision-language models

## Abstract

Recent advances in large language models (LLMs) and vision transformers have enabled multimodal systems that integrate clinical text with medical imaging for diagnostic decision-making. While these systems show promising results on benchmark datasets in well-resourced research settings, their applicability in low-resource healthcare environments where diagnostic disparities are most severe remains limited and poorly understood. This mini review synthesizes key developments in LLM–vision fusion architectures from 2018 to 2026, with a focus on radiology-oriented visual question answering (VQA) and report generation systems viewed from a deployment perspective. Rather than comprehensively cataloguing multimodal medical AI, we synthesize the evolution of LLM–vision fusion architectures and discuss complementary deployment-enabling strategies, including parameter-efficient adaptation, post-training quantization, federated learning, and multilingual support, where they directly improve the feasibility of radiology AI in resource-constrained healthcare settings. Rather than focusing solely on performance benchmarks, we examine these approaches through a deployment-oriented lens, highlighting trade-offs between representational capacity, computational efficiency, interpretability, and memory footprint. We argue that current progress remains substantially shaped by model scaling and benchmark optimization, which often do not address the memory, connectivity, and annotation constraints of low-resource healthcare systems. While cross-modal transformer architectures provide strong representational alignment, their computational demands and reliance on large curated datasets limit real-world deployment. In contrast, emerging directions including parameter-efficient fine-tuning, post-training quantization, federated learning, and modular agent-based systems offer more tractable pathways toward clinical integration under hardware and data constraints. To bridge the gap between benchmark performance and clinical utility, we identify concrete challenges in data scarcity, multilingual coverage, and calibration, and propose a shift toward lightweight, interpretable, and hardware-aware multimodal AI. This perspective highlights the need to move beyond scaling-centric design toward models that can run on 4–8 GB VRAM, operate offline, and generalize across languages and imaging equipment.

## 1 Introduction

Low-resource healthcare systems continue to face significant structural challenges that limit effective care delivery. These environments are often characterized by shortages of trained medical professionals, fragmented patient records, and inadequate diagnostic infrastructure. The World Health Organization projects a global shortage of approximately 11 million health and care workers by 2030, disproportionately affecting low- and middle-income countries (1). In parallel, clinical data is frequently dispersed across paper-based or poorly integrated digital systems, resulting in incomplete patient profiles and delayed diagnostic processes and significant clinical bottlenecks (2).

Deploying AI solutions in healthcare often requires addressing a broad spectrum of resource-related constraints. These include limited computational capacity and unreliable electricity or internet connectivity; economic barriers in low-income countries; clinical workforce shortages falling below WHO-recommended thresholds; scarcity of annotated medical datasets; the need to support non-English clinical workflows; and heterogeneous imaging equipment lacking standardization. While the reviewed literature addresses some of these dimensions more comprehensively than others, this review considers systems relevant to deployment under all of these constraints.

In contrast, high-resource healthcare systems have increasingly adopted advanced artificial intelligence (AI) technologies to support complex diagnostic tasks. Multimodal AI systems that integrate imaging data with clinical text and patient history have demonstrated substantial improvements in diagnostic accuracy and workflow efficiency. However, the benefits of these systems remain unevenly distributed. In low-resource settings, adoption is constrained by infrastructural limitations, data scarcity, and a fundamental mismatch between model design and real-world clinical conditions, thereby reinforcing existing disparities in healthcare access and quality (3).

Early medical AI systems were predominantly unimodal, focusing either on imaging or text-based data. Convolutional neural networks, including ResNet and DenseNet, achieved strong performance in tasks such as lesion detection and image segmentation (4, 5), while transformer-based models such as BioBERT (6) and ClinicalBERT (7) advanced clinical natural language processing for tasks including medical note classification and question answering. Despite these successes, unimodal systems remain inherently limited, as clinical diagnosis requires the integration of heterogeneous data sources, including imaging findings, laboratory results, and patient history (8).

Recent multimodal LLM–vision models enable deeper interaction between clinical text and imaging. Early approaches adapted general-domain visual question answering (VQA) frameworks and attention-based fusion mechanisms for initial medical exploration (9). Subsequent developments introduced contrastive vision–language pretraining to improve cross-modal alignment and enable zero-shot transfer (10). More recent architectures leverage cross-modal transformers and instruction tuning to support structured reasoning on clinically relevant tasks (11).

Despite rapid progress, current research remains heavily oriented toward scaling model size and optimizing benchmark scores. Such approaches are often misaligned with the constraints of low-resource healthcare systems, where GPU memory is limited to 4–8 GB, annotated data are scarce, and internet connectivity is intermittent. Moreover, reliance on curated datasets and controlled evaluation settings reduces clinical validity, while hallucination, domain shift, and limited interpretability further restrict applicability (3). These limitations highlight a critical gap between benchmark performance and deployment feasibility.

Rather than offering an exhaustive survey, this review centers on radiology-focused vision–language architectures for VQA, visual dialog, report generation, and related image–text interpretation tasks, examined against deployment criteria relevant to low-resource healthcare, including parameter count, inference memory, offline operability, language coverage, and clinical validation. The core radiology evidence is supplemented by a limited number of broader multimodal medical foundation models and agentic systems that provide relevant architectural or benchmark comparators, together with deployment-enabling studies on quantization, federated learning, parameter-efficient adaptation, and multilingual support. These non-core studies are explicitly identified and are not presented as direct evidence of radiology-specific clinical performance. We analyze the evolution of multimodal architectures, examine trade-offs between performance, memory efficiency, and interpretability, and evaluate emerging strategies such as parameter-efficient fine-tuning, post-training quantization, and federated learning. We argue that current progress remains substantially shaped by model scaling and benchmark optimization, which often do not address the memory, connectivity, and annotation constraints of low-resource healthcare systems.

To support this perspective, we propose a conceptual rather than empirically validated framework that follows a contemporary VLM development-to-application pipeline: multimodal data fusion and pre-training; fine-tuning and adaptation; resource-aware optimization and deployment; and, finally, agentic orchestration and clinician-supervised decision support at the application layer.

### 1.1 Literature search and study selection

This mini review was conducted as a narrative rather than systematic review. Relevant studies were identified through searches of PubMed, IEEE Xplore, Scopus, ACM Digital Library, and Google Scholar. Search terms included combinations of “multimodal medical diagnosis,” “LLM–vision fusion,” “medical visual question answering,” “radiology report generation,” “vision-language model,” “low-resource healthcare AI,” “federated learning medical imaging,” and “model quantization clinical AI.” The search covered publications from January 2018 to March 2026.

Studies were classified through three explicit inclusion pathways. Tier 1 (core radiology studies; n=13) included multimodal architectures that combined radiological images, including chest radiography, CT, MRI, or mixed radiological imaging, with clinical text or VQA pairs and evaluated radiology-relevant VQA, visual dialog, report generation, classification, or image–text interpretation. Tier 2 (broader multimodal or agentic comparators; n=7) included general biomedical foundation models and multi-agent systems that were not radiology-exclusive but were retained when their architecture, benchmark performance, or computational requirements established a relevant comparison point or an orchestration pattern transferable to radiology deployment. These studies were treated as architectural or benchmark context, not as direct evidence of radiology-specific clinical performance. Tier 3 (deployment-enabling technique studies; n=3) included work on quantization, parameter-efficient adaptation, and multilingual VQA when the demonstrated technique directly addressed a computational, annotation, or language constraint relevant to low-resource radiology AI, even if the original evaluation used another medical modality or a non-radiology task. Radiology-specific federated report generation remained in Tier 1 because its original task and datasets met the core radiology criterion.

Studies were excluded if they did not satisfy any of these three pathways, lacked sufficient architectural or empirical detail for comparative assessment, or addressed unimodal or non-medical tasks without a directly transferable deployment contribution. Quantitative evaluation was required for core radiology studies. Broader comparator or deployment-enabling papers with qualitative or non-radiology evaluation were retained only when their contextual role was explicitly identified in Table 1.

**Table 1:** Comparative summary of 23 representative studies relevant to multimodal radiology AI and low-resource deployment (2018–2026).

Study	Year	Task	Arch.	Params	GPU/RAM	Open	Offline	Lang.	Valid.	Performance measure and reported result	Deployment limit
*Cluster A foundational VQA & hybrid fusion (2018–2021)*											
Lau et al. (12)	2018	Radiology VQA	SAN/MCB (VQA-RAD)	∼50M	1 GPU; ∼4 GB VRAM	Open	Yes	EN only	Benchmark	Acc: 60.6% closed; 25.4% open-ended	Small dataset (315 images); weak open-ended performance
Kovaleva et al. (13)	2020	Radiology Visual Dialog	Stacked Attn (SAN) (MIMIC-CXR (36))	∼110M	1–2 GPU; ∼8 GB	Open	Yes	EN only	Benchmark	MRR: 0.41 (silver); 0.46 (gold labels)	Heavy class imbalance; dialog format limits direct radiology VQA transfer
Sharma et al. (14)	2021	Medical VQA	MFB + Co-Attention (ImageCLEF)	∼85M	1 GPU; ∼6 GB	Open	Yes	EN only	Benchmark	Acc: 78.4% (closed VQA)	Limited annotated training data; shallow cross-modal interaction
Ren & Zhou (15)	2020	VQA & Gen	Transformer (Masked) (ImageCLEF)	6.4M	1 GPU; ∼2 GB	Open	Yes	EN only	Benchmark	Acc: 74.1% (VQA); BLEU-1: 0.312 (Gen)	Lowest parameter count reviewed; limited generative accuracy
*Cluster B report generation & clinical memory (2021–2025)*											
Chen et al. (20)	2021	Report Generation	R2Gen (Relational Memory) (IU-Xray/MIMIC-CXR (36))	∼110M	1 GPU; ∼8 GB	Open	Yes	EN only	Benchmark	IU: BLEU-4 = 0.165, ROUGE-L = 0.383; MIMIC: BLEU-4 = 0.103, ROUGE-L = 0.277	Fixed memory matrix; no patient-history integration
Sultan et al. (37)	2025	Report Summarization	T5 + ViT (Cross-Attn) (IU Chest X-rays)	∼220M	RTX 4070 Laptop; 16 GB	Open	Yes	EN only	Benchmark	BLEU: 0.847; ROUGE-L: 0.851	220M params; limited incidental finding coverage
Islam et al. (21)	2025	Report Generation	ViT-GPT2/Swin-BART (IU-Xray)	∼124M	T4 GPU; ∼16 GB	Open	Yes	EN only	Benchmark	BLEU: 0.040; ROUGE-L: 0.203	Sensitive to low-resolution or noisy images common in LMICs
Ansoy et al. (19)	2025	Report Generation	ViT-GPT2 + MedVAG (Graph) (IU-Xray/COV-CTR)	∼180M	1–2 GPU; ∼24 GB	Open	Yes	EN only	Benchmark	IU: BLEU-4 = 0.183, ROUGE-L = 0.371; COV: BLEU-4 = 0.201, ROUGE-L = 0.412	Graph fusion expensive; ∼24 GB VRAM required
Bannur et al. (26)	2024	Report Generation	MAIRA-2 (grounded) (MIMIC-CXR (36))	7B/13B	A100 80 GB+	Weights	Cond.	EN only	Benchmark	RadFact logical precision: 52.5% (7B), 55.6% (13B); RadGraph-F1: 34.6% (7B), 35.9% (13B), MIMIC-CXR	A100 80 GB required; dense grounding annotations infeasible in LMICs
*Cluster C contrastive pretraining & grounded MLLMs (2022–2026)*											
Wang et al. (16)	2022	Medical CLIP/Zero-shot	MedCLIP (MIMIC/CheXpert)	∼86M	1 GPU (RTX 3090); 24 GB	Open	Yes	EN only	Benchmark	AUC: 0.878 (CheXpert supervised); Zero-shot AUC: 0.821	UMLS dependency; zero-shot degrades on non-standard terminology
Zhang et al. (25)${}^{†}$	2023	Biomedical image–text pretraining; broad biomedical modalities	BioMedCLIP (PubMed 15M)	NR (ViT-B/16 + PubMedBERT)	Up to 16×A100/V100 (training)	Open	Yes	EN only	Benchmark	Zero-shot AUROC: 67.04% (TCGA-TIL); zero-shot accuracy: 78.95% (RSNA)	15M-pair pretraining not replicable in LMICs; EN-centric dataset
Li et al. (17)${}^{†}$	2023	Biomedical VQA; mixed biomedical pretraining with radiology subsets	Projector + Vicuna LM (PMC/VQA-RAD/SLAKE)	7B	8 × A100 train; 1 × A100 inf.	Open	Yes	EN only	Benchmark	Accuracy (closed-ended, 7B): VQA-RAD 84.19%; SLAKE 85.34%	8 × A100 for training; hallucination risk in low-oversight settings
Moor et al. (22)${}^{†}$	2023	Few-shot generative medical VQA; not radiology-exclusive	Med-Flamingo (MTB pretraining; VQA-RAD/PathVQA/Visual USMLE)	8.3B	A100 (8.3B inf.)	Open	Yes	EN only	Benchmark	Clinical evaluation score (0–10): 5.61 (VQA-RAD, 6-shot), 4.33 (Visual USMLE, 4-shot); exact match: 0.303 (PathVQA, 6-shot)	8.3B params; A100 required; hallucination risk on sparse context
Tu et al. (27)${}^{†}$	2023	Multitask multimodal medical QA; radiology among broader tasks	Med-PaLM M (PaLM-E) (MMB)	Up to 562B	TPU pod (closed)	Closed	No	EN only	Benchmark	BLEU-1/F1 (562B): VQA-RAD 71.27%/62.06%; SLAKE 92.70%/89.28%	Up to 562B params; closed infrastructure; incompatible with offline LMIC use
Wu et al. (28)${}^{†}$	2023	Qualitative medical-image evaluation; not radiology-exclusive	GPT-4V (closed API) (multi-modality)	NR (closed)	API only	Closed	No	EN only	Qualitative only	NR	Closed-source; API-dependent; no offline or privacy-preserving use
*Cluster D agentic & multi-agent frameworks (2024–2025)*											
Chen et al. (29)	2024	Chest X-ray interpretation benchmark	CheXagent (Foundation) (CheXbench)	∼8B	A100 (∼8B inf.)	Open	Cond.	EN only	Benchmark	Accuracy: view classification 97.5% (MIMIC-CXR), VQA 78.1% (SLAKE); RadGraph score: 18.6 (MIMIC-CXR findings generation)	8B params; A100 required; no offline validation
Zhou et al. (23)${}^{†}$	2025	Multi-agent diagnosis across image, text, audio, and video	Modular LLM Collaboration (PMC-VQA/MedVidQA)	Multi-LLM	API-dependent	NR	No	EN only	Benchmark	Accuracy: PMC-VQA 32.5%; MedVidQA 74.3%	API-dependent; coordination overhead; no offline path
Liu et al. (24)${}^{†}$	2025	Glaucoma diagnosis from fundus imaging; agentic comparator	Vision + Multi-LLM Agents (Harvard-FairVision)	NR	API-dependent	NR	No	EN only	Benchmark	AUC: 0.941; accuracy: 88.7%	API-dependent; no domain-specific fine-tuning; no clinical validation
*Cluster E resource-aware optimisation & deployment (2019–2025)*											
Kumar et al. (18)	2025	Tuberculosis chest-X-ray classification	Cross-Modal Transformer (Govt. Medical College, India)	∼125M	CPU workstation (64 GB RAM)	NR	Yes	EN only	Retro.	Accuracy: 96.2%; F1-score: 0.961; AUC: 0.982	Single-institution dataset; limited imaging diversity
El Mir et al. (34)${}^{‡}$	2025	Medical VQA/Summ.	Fine-tuning + PTQ (4/8-bit) (VQA-RAD; SLAKE; PathVQA)	7B → 0.9B	Dynamic memory: 5.18 GB (4-bit LLaVA-Med); 1.68 GB (FQ4)	Open	Yes	EN only	Benchmark	Accuracy (closed-ended VQA-RAD, 4-bit): 82.1%; change in open-ended PathVQA accuracy vs. FP: −6.3 percentage points	Open-ended accuracy drops under quantization; EN-only benchmarks
Bhuyan et al. (35)${}^{‡}$	2025	Bengali VQA on non-radiology medical images; multilingual comparator	Multi-head Attn + Gated Fusion (BVQA, Bangladesh)	∼110M	1 GPU (V100); 16 GB	Open	Yes	Bengali	Benchmark	Accuracy: 74.7%	Bengali only; single-word answers; no radiology images
Hasan et al. (30)	2025	Federated Report Gen.	ViT-GPT2 + Federated Learning (IU-Chest/MIMIC (36))	∼124M	1 GPU per node; ∼16 GB	Open	Yes	EN only	Benchmark	BLEU: 0.040; ROUGE-L: 0.207 (federated)	Communication overhead; heterogeneous nodes reduce convergence
Houlsby et al. (32)${}^{‡}$	2019	Adapter-based parameter-efficient fine-tuning; non-medical NLP	Adapter Modules (GLUE, 26 tasks)	∼0.9M add-on; 3%–4% trainable	4 TPUs	Open	Yes	EN only	Benchmark	GLUE average score: 80.0% with adapters vs. 80.4% with full fine-tuning	0.4-point accuracy gap vs full fine-tuning; NLP only

Marked studies are included for an explicitly stated comparative or methodological purpose and are not treated as direct evidence of radiology-specific clinical performance. Performance measures and results are reproduced from the corresponding source studies and should not be interpreted as direct cross-study rankings because datasets, tasks, splits, and evaluation protocols are heterogeneous.

The performance column identifies the named measure before its corresponding result. Comparative outcomes without a reported aggregate value are explicitly labeled NR. Hardware, memory, parameter-efficiency, validation status, API dependency, and qualitative-evaluation information are reported in their corresponding columns rather than being presented as performance measures.

NR, Not Reported; FP, Full Precision; PTQ, Post-Training Quantization.

Open-source: Open, publicly available code and weights; Weights, pretrained weights only; Closed, proprietary.

Offline: Yes, local inference supported; Cond., possible with additional adaptation; No, cloud/API required.

Clin. Valid.: Benchmark, public benchmark only; Retro., retrospective clinical evaluation; Qualitative only, no quantitative benchmark value reported.

Unmarked, Tier 1 core radiology study (n=13);

${}^{†}$Tier 2 broader multimodal or agentic comparator (n=7);

${}^{‡}$Tier 3 deployment-enabling technique study (n=3).

From an initial pool of approximately 180 candidate papers identified across databases, 23 studies met one of the three predefined inclusion pathways and were included in the comparative evidence synthesis (Table 1): 13 Tier 1 studies, 7 Tier 2 studies, and 3 Tier 3 studies. Tier 2 and Tier 3 entries are identified by † and ‡, respectively, in Table 1; unmarked entries are Tier 1 studies. In addition, 11 contextual or methodological references were used solely to support background concepts, benchmark or dataset provenance, methodological foundations, and deployment considerations; these were not part of the tiered comparative evidence set and are not counted among the included studies. All 11 contextual references and their specific roles are identified explicitly in Supplementary Table S1. Selection was guided by architectural coverage, recency, publication relevance, and direct applicability to the computational, connectivity, annotation, language, privacy, or clinical-validation constraints of low- and middle-income country (LMIC) settings. We acknowledge that this narrative process does not eliminate selection bias; high-profile English-language papers from high-income research groups are likely overrepresented relative to grey literature and non-English sources.

## 2 Evolution of multimodal medical AI (2018–2026)

Within this radiology-centered but explicitly stratified scope, multimodal medical AI has evolved from task-specific fusion models to systems supporting contrastive alignment, instruction tuning, structured generation, agentic coordination, and hardware-aware deployment. Core radiology studies provide the primary evidence for VQA, visual dialog, report generation, and related image–text interpretation. Broader biomedical foundation models and multi-agent systems are discussed as clearly labeled comparators where they illuminate transferable architectural patterns or establish a performance–hardware reference point. Deployment-enabling studies are included where they directly inform the feasibility of adapting or operating radiology VLMs under constrained memory, connectivity, privacy, annotation, or language conditions.

### 2.1 Foundations and representation learning (VQA and instruction-tuned models)

Early multimodal approaches were primarily centered on VQA and the incorporation of clinical context into imaging tasks. Lau et al. (12) demonstrated the importance of domain-specific datasets through VQA-RAD, while Kovaleva et al. (13) showed that integrating patient history via attention mechanisms reduces ambiguity in chest X-ray interpretation.

To address computational constraints, efficient fusion techniques such as Multimodal Factorized Bilinear Pooling (MFB) (14) and parameter-reduced transformer architectures (15) were introduced, enabling training under limited hardware. However, these models relied on shallow cross-modal interactions that preclude joint early-layer reasoning.

Dataset scarcity further motivated contrastive representation learning. MedCLIP (16) improved cross-modal alignment and enabled zero-shot transfer without task-specific annotation, while instruction-tuned systems such as LLaVA-Med (17) provided more flexible biomedical reasoning at the cost of requiring 8 × A100 GPUs for training.

### 2.2 Advanced reasoning and agent-based systems

Subsequent work focused on cross-modal reasoning through deeper architectural integration. Cross-modal transformer models (18) enable bidirectional attention between image patches and text tokens, improving semantic alignment and contextual grounding. Memory-augmented architectures, including MedVAG (19) and R2Gen (20), improve report coherence by tracking salient visual findings across decoding steps.

Lightweight encoder–decoder models such as ViT–GPT2 (21) demonstrate that competitive report generation is achievable on a consumer T4 GPU (∼16 GB VRAM), a practically significant finding for LMIC deployment. Med-Flamingo (22) illustrates few-shot adaptation under annotation scarcity, though its 8.3B parameter count still requires an A100 for inference.

More recent research introduces agent-based systems that decompose diagnostic reasoning into coordinated, role-specific components. Multi-agent frameworks (23) and systems such as MedChat (24) enable collaborative decision-making by integrating specialized agents and external tools. These approaches improve interpretability by exposing intermediate reasoning steps, but introduce coordination overhead and inter-module error propagation.

Recent models have significantly advanced multimodal benchmark capability, though their feasibility for offline or resource-constrained deployment varies considerably. BioMedCLIP (25) scales contrastive pretraining to 15 million scientific image–text pairs; however, its pretraining requirement of up to 16 A100 or V100 GPUs is not replicable in most LMIC contexts. MAIRA-2 (26) introduces phrase-level anatomical grounding for report generation, improving output traceability; its A100-class memory requirement makes it infeasible for clinic-grade hardware. Med-PaLM M (27) scales to 562B parameters on closed TPU infrastructure, while GPT-4V (28) is available only through a closed API and provides qualitative rather than directly comparable quantitative evidence in the cited study. CheXagent (29) provides a radiology-specific instruction-tuned foundation model with structured chest X-ray benchmarking; its 8B parameter size and accelerator requirement reflect the same infrastructure gap. Collectively, these models confirm that broad or highly grounded multimodal capability and sub-8 GB local inference remain in tension, reinforcing the case for the quantization, parameter-efficient fine-tuning, and federated approaches reviewed in Section 2.3.

### 2.3 Deployment-enabling techniques for radiology AI: privacy, efficiency, and quantization

The techniques reviewed in this subsection federated learning, parameter-efficient fine-tuning, post-training quantization, and multilingual dataset construction are deployment enablers rather than architectural contributions to radiology VQA or report generation per se. They make it feasible to run, adapt, or train radiology LLM–vision systems under the memory, connectivity, and annotation constraints of low-resource environments. Their inclusion is justified by this deployment relevance.

Federated learning frameworks (30, 31) enable collaborative training across institutions without centralizing sensitive patient data, a critical property where data-sharing agreements are absent. Parameter-efficient adaptation via adapter modules (32) and low-rank fine-tuning (33) reduces the number of trainable parameters to 1%–4% of the backbone, allowing domain adaptation on a single mid-range GPU.

Post-training quantization (34) reduces model memory by 65%–89%, enabling inference on RTX 3050 (6 GB) or Jetson AGX Orin class hardware. Localized multilingual datasets such as BVQA (35) demonstrate that language adaptation is achievable under constrained annotation budgets, though clinical validation in those languages remains an open problem.

## 3 Comparative analysis of LLM–vision fusion architectures

We compare 23 representative studies across three explicitly labeled inclusion pathways: 13 core radiology studies (Tier 1), 7 broader multimodal or agentic comparators (Tier 2; marked †), and 3 deployment-enabling technique studies (Tier 3; marked ‡). The analysis emphasizes inference hardware and memory, offline operability, language coverage, clinical validation, named performance measures, and their corresponding reported results. Studies marked † or ‡ are not interpreted as radiology-specific clinical evidence; they are included only where they provide a relevant architectural comparator or demonstrate a transferable deployment strategy.

Table 1 summarizes these studies. Because datasets, tasks, splits, and evaluation protocols differ substantially, the reported values should not be interpreted as direct rankings across studies. NR denotes not reported, FP denotes full precision, and PTQ denotes post-training quantization.

The table reveals two dominant patterns. First, large benchmark-oriented or highly grounded systems such as Med-PaLM M and MAIRA-2 require accelerator-class infrastructure, while GPT-4V depends on a closed cloud API and does not report a directly comparable quantitative result in the cited study. These profiles place all three outside the targeted offline LMIC deployment setting. Second, models that fit within a single consumer GPU (ViT–GPT2 at ∼16 GB; quantized LLaVA-Med at 5.18 GB; TinyLLaVA-Med-FQ4 at 1.68 GB) demonstrate local feasibility, but heterogeneous tasks and evaluation protocols prevent a direct score ranking against the larger systems. The recurring tension is therefore between computational accessibility, output complexity, and benchmark capability rather than a single universal hardware–accuracy curve.

The reviewed literature organises naturally into four architectural families, each reflecting a distinct point on the trade-off between representational capacity, inference memory, and low-resource deployability.

### 3.1 Early fusion architectures: bilinear pooling and co-attention

Early multimodal approaches encoded each modality with a separate unimodal encoder, then combined the resulting vectors through a learned interaction module. Two strategies dominated. *Bilinear pooling* most notably MFB (14) compresses the outer product of image and text features into a fixed-size vector, capturing multiplicative cross-modal correlations without full pairwise attention. *Stacked attention networks* (SAN) (12, 13) alternately attend over image regions conditioned on the query and over query tokens conditioned on the image. The defining advantage of this family is low memory: Ren and Zhou (15) achieved 74.1% VQA accuracy with only 6.4M parameters on a single GPU with ∼2 GB VRAM, confirming that shallow fusion can operate under tight hardware budgets. The corresponding limitation is that modality interaction occurs only at the final representation stage; the model cannot resolve ambiguities requiring joint reasoning across modalities (e.g., linking a specific lung region in an X-ray to a negation in the accompanying clinical note). This bottleneck motivated the shift to deep cross-modal transformers.

Across this cluster, parameter count and reported memory do not increase in a strictly proportional manner. The four systems span approximately 6.4M–110M parameters and approximately 2–8 GB of reported GPU memory, while the directly reported VQA accuracies range from 60.6% to 78.4%. Ren and Zhou’s 6.4M-parameter model and Sharma et al.’s approximately 85M-parameter model both remain within a sub-6 GB profile, suggesting that fusion dimensionality, image resolution, intermediate feature maps, and implementation choices can be as important to memory demand as nominal parameter count. Because the studies use different datasets and hardware-reporting conventions, this pattern is an association across studies rather than a causal or head-to-head comparison.

### 3.2 Deep cross-modal reasoning: transformers, memory, and report generation

Cross-modal transformers interleave image patch tokens and text tokens within the same attention layers, allowing each modality to condition the other from the first representation layer. Kumar et al. (18) applied this to TB classification, achieving 96.2% accuracy and AUC 0.982 on a CPU workstation (64 GB RAM), a hardware profile accessible in district hospitals. For report generation, the challenge is producing multi-sentence outputs that remain internally consistent across anatomical regions. Two mechanisms address this. R2Gen (20) maintains a relational memory matrix that retrieves salient visual findings at each decoding step (IU-Xray BLEU-4: 0.165; ROUGE-L: 0.383), operating within a single patient encounter rather than across visits. MedVAG (19) constructs a graph over multi-view radiograph features and uses cross-attention to propagate findings across views (IU-Xray BLEU-4: 0.183; COV-CTR BLEU-4: 0.201), at the cost of ∼24 GB VRAM. ViT–GPT2 (21) (∼124M parameters, T4 GPU) provides a lighter report-generation option (IU-Xray ROUGE-L: 0.203), while MAIRA-2 (26), which adds phrase-level anatomical grounding and richer contextual inputs, requires A100-class hardware despite improved output traceability.

Taken together, these studies show a recurring trade-off between output structure and computational cost. Lightweight encoder–decoder systems generating free text operate on approximately 8–16 GB devices, whereas graph-based cross-view reasoning and phrase-level anatomical grounding are reported with substantially higher memory requirements. Model scale is an important confounder, particularly for MAIRA-2, so the available evidence does not isolate grounding as the sole cause of the additional cost. Nevertheless, the family-level pattern is consistent: richer cross-view structure, anatomical grounding, and output traceability tend to be obtained at the expense of higher inference memory and implementation complexity.

### 3.3 Contrastive alignment and instruction-tuned MLLMs

This family shapes the shared embedding space directly rather than relying solely on supervised cross-entropy loss. *Contrastive alignment* maximises similarity between matched image–text pairs and repels unmatched ones, enabling zero-shot transfer without task-specific annotation. MedCLIP (16) achieved zero-shot AUC of 0.821 on CheXpert (38) using UMLS-guided unpaired contrastive learning on a single RTX 3090 (24 GB). BioMedCLIP (25) extended pretraining to 15 million PubMed image–text pairs and demonstrated zero-shot performance across mixed biomedical image types; its pretraining scale of up to 16 A100 or V100 GPUs is not independently reproducible in most LMIC settings. *Instruction tuning* (11) fine-tunes a pretrained language backbone on curated instruction–response pairs to produce structured clinical outputs. LLaVA-Med (17), derived from the LLaVA framework (39), reports closed-ended accuracy of 84.19% on VQA-RAD and 85.34% on SLAKE for a 7B variant but requires 8×A100s for training. Med-Flamingo (22) (8.3B parameters) enables few-shot generative medical VQA, reporting a clinician-rated score of 5.61/10 on VQA-RAD in the 6-shot setting, at the cost of A100-class inference. At the upper scale bound, Med-PaLM M (27) reports strong BLEU-1 and F1 results across medical VQA tasks but requires closed TPU infrastructure, while the cited GPT-4V study (28) provides qualitative case evaluations without a comparable aggregate benchmark. The pattern across this cluster is consistent: contrastive and instruction-tuned models reduce annotation requirements, which partially aligns with low-resource constraints, but the infrastructure demands of the largest models can negate this benefit.

A cross-study comparison further shows that annotation efficiency and deployment efficiency are distinct properties. MedCLIP reduces dependence on task-specific labels while remaining within a single 24 GB GPU profile, whereas larger instruction-tuned and few-shot systems require A100-class hardware despite also reducing task-specific annotation requirements. At the upper scale bound, closed models shift inference cost to remote cloud infrastructure rather than eliminating it. Consequently, efficiency claims in this family should distinguish reduced supervision requirements from local inference feasibility; improvement in one does not imply improvement in the other.

### 3.4 Agentic, retrieval-augmented, and resource-aware systems

The most recent family comprises both coordinated agent pipelines and resource-aware deployment methods, but these subfamilies address different constraints. MAM (23) assigns role-specialized LLMs to diagnostic subtasks and reports accuracy of 32.5% on PMC-VQA and 74.3% on MedVidQA. Because these benchmarks extend beyond radiology-specific VQA, MAM is included as a transferable agentic comparator rather than as radiology-specific evidence. MedChat (24) applies specialist agents to glaucoma diagnosis using fundus imaging and is treated similarly. CheXagent (29) is a radiology-specific instruction-tuned foundation model evaluated through task-specific CheXbench measures; representative results include 97.5% accuracy for MIMIC-CXR view classification, 78.1% accuracy for SLAKE VQA, and a RadGraph score of 18.6 for MIMIC-CXR findings generation. None of these systems has undergone prospective clinical validation in an LMIC setting.

The resource-aware subfamily instead treats memory, trainable-parameter count, privacy, and connectivity as first-class constraints. El Mir et al. (34) quantized LLaVA-Med to 4-bit, reducing dynamic memory from 14.86 GB to 5.18 GB, and reduced a fine-tuned TinyLLaVA-Med variant to 1.68 GB. The 4-bit model reports 82.1% closed-ended VQA-RAD accuracy, while open-ended PathVQA accuracy decreases by 6.3 percentage points relative to full precision. Adapter modules (32) and LoRA (33) reduce the trainable share of the backbone, federated ViT–GPT2 (30, 31) avoids centralizing institutional data, and BVQA (35) demonstrates Bengali-language adaptation on a non-radiology benchmark.

Comparing the two subfamilies reveals a shared unresolved gap. Agentic systems add role specialization, routing, retrieval, or collaborative reasoning, improving task decomposition and potential traceability but increasing coordination complexity and external-model dependency. Quantization, PEFT, and federated learning reduce memory, adaptation, or data-centralization requirements without independently supplying an agentic application layer. No reviewed system combines grounded agentic orchestration, sub-8 GB local inference, fully offline operation, and prospective radiology-specific validation in one platform. This missing combination motivates the staged conceptual framework in Section 5.

## 4 Challenges and future research directions in low-resource healthcare deployment

The deployment of multimodal medical AI in low-resource settings faces interconnected challenges. The subsections below move from hardware constraints to data quality, language coverage, clinical validation, and governance a sequence that reflects the order in which these barriers are encountered during deployment.

### 4.1 Hardware optimization and model quantization

Most reviewed models require NVIDIA A100 or H100 GPUs for inference hardware absent from virtually all LMIC clinic settings. The quantization results of El Mir et al. (34) provide the clearest evidence of what is achievable on constrained hardware: 4-bit PTQ of LLaVA-Med fits into 5.18 GB VRAM (RTX 3050 class), and 4-bit quantization of fine-tuned TinyLLaVA-Med requires only 1.68 GB (Jetson AGX Orin class), with closed-ended VQA accuracy drops of 2.6 and 0 percentage points respectively. Open-ended generation degrades more substantially under quantization, indicating that diagnostic text generation requires higher numerical precision than answer classification. ViT–GPT2 (21) (∼124M parameters, ∼16 GB VRAM) demonstrates that a non-quantized lightweight architecture can match much larger report generation models on IU-Xray, though its sensitivity to low-resolution inputs common in LMIC imaging limits applicability without preprocessing pipelines. Future work should pursue three concrete directions: (1) systematic benchmarking of 4-bit and 8-bit PTQ models on radiology-specific tasks (not general VQA) using devices representative of district hospital hardware in sub-Saharan Africa and South Asia (4–8 GB VRAM); (2) inference pipelines with fully offline operation, local output storage during connectivity gaps, and secure synchronization when bandwidth is available; and (3) hardware-aware neural architecture search targeting the memory and compute budgets of edge devices (Jetson AGX Orin, Raspberry Pi 5) rather than compressing models originally designed for A100 clusters.

### 4.2 Data scarcity, bias, and domain shift

Low-resource institutions produce heterogeneous data: low-resolution images from older equipment, scanned paper reports, and handwritten clinical notes. Models trained on high-income radiology datasets (MIMIC-CXR, CheXpert) systematically misclassify conditions endemic to LMIC populations, as demonstrated for chest radiographs by Seyyed-Kalantari et al. (40). ViT–GPT2 is particularly sensitive to input resolution degradation (21). Synthetic augmentation can partially offset annotation scarcity but risks amplifying existing demographic and equipment biases if the generative model was trained on the same skewed data (40). Few-shot methods such as Med-Flamingo (22) and contrastive pretraining via MedCLIP (16) reduce annotation requirements but do not address model calibration on out-of-distribution populations. Future work should develop and evaluate domain-shift calibration methods temperature scaling, histogram-based recalibration, conformal prediction applied to radiology VQA and report generation models before deployment at new clinical sites, with calibration assessed on local data (local equipment, local disease prevalence, local imaging protocols) rather than held-out benchmark splits. Uncertainty estimates should be surfaced to clinicians rather than suppressed, and evaluation should measure agreement with local radiologist reference diagnoses rather than curated dataset labels.

### 4.3 Multilingual adaptation

Every model in Table 1 is developed primarily in English except BVQA (35), which covers Bengali but is limited to single-word answers on non-radiology images. Parameter-efficient fine-tuning (PEFT), including LoRA (32, 33) provides a practical route to language adaptation without full retraining. However, language adaptation without in-language clinical validation is insufficient because performance on translated benchmarks may not generalize to authentic clinical terminology or diagnostic reasoning. Future work should prioritize the development of radiology VQA and report generation datasets in underrepresented languages (e.g., Bengali, Hindi, Swahili, and Amharic), followed by prospective validation using institution-specific reference diagnoses.

### 4.4 Clinical validation and regulatory compliance

BLEU and ROUGE scores measure n-gram overlap with reference reports; they do not measure diagnostic correctness, finding sensitivity, or specificity. No model reviewed in this paper has been validated in a prospective clinical study in a low-resource setting, and only one [Kumar et al. (18)] reports any retrospective clinical evaluation. This is the most critical gap between the reviewed literature and real deployment. Validation is further complicated by absent regulatory frameworks: the FDA Software as a Medical Device pathway and EU CE marking process require infrastructure and dossier resources unavailable in most LMICs, yet most low-income country regulators have no AI-specific approval guidelines. This creates a deadlock deployment requires approval; approval requires deployment evidence. Future validation work should adopt clinician-reader study designs in which clinicians at low-resource sites interpret radiographs with and without AI assistance, with outcomes measured against specialist consensus or follow-up data. Studies should report sensitivity and specificity on clinically relevant findings, reader agreement (Cohen’s κ), and time-to-diagnosis, powered for local disease prevalence and conducted in the working language of the site.

### 4.5 Privacy-preserving learning and governance considerations

Federated learning (30, 31) enables cross-institution training without centralizing patient records, a critical property where formal data-sharing agreements are absent or unenforceable. Practical barriers remain: communication overhead between federation nodes is substantial at the kilobit-per-second uplink speeds typical of district hospitals, and data heterogeneity across nodes different imaging equipment, patient populations, disease prevalence slows and destabilizes convergence. Future federated work should target communication-efficient strategies (gradient compression, asynchronous aggregation, hierarchical federation) suited to low-bandwidth settings, and should establish federated evaluation protocols that measure site-level performance variance and demographic fairness, not only aggregate accuracy. Beyond federated training, three governance problems lack adequate solutions. First, model hallucination and overconfidence (3, 17) require mandatory human-in-the-loop review before any diagnostic output is acted on; autonomous deployment is not appropriate in settings where oversight is scarce. Second, high-income training datasets (MIMIC-CXR (36), PMC) are routinely reused in LMIC contexts without explicit patient re-consent, raising unresolved data ownership questions. Third, liability for diagnostic errors by AI systems is legally undefined in most low-resource countries. These gaps must be addressed through policy development alongside technical research, not after deployment.

### 4.6 Fundamental limitations of the current evidence base

The limitations of this review reflect limitations of the field. Selection bias toward high-profile English-language papers from high-income research groups is likely; grey literature and non-English sources are underrepresented. Nearly all reviewed studies report benchmark performance on curated English-language datasets under controlled conditions; real-world deployment evidence from LMIC settings is almost entirely absent. Publication bias favors positive results, leaving the failure modes of these systems undocumented. Clinician and patient perspectives from resource-limited settings are largely absent from technical literature. These are not flaws in individual papers but structural features of the research ecosystem that limit how broadly current findings can be applied.

## 5 Conceptual framework for multimodal diagnosis

**Figure 1:**
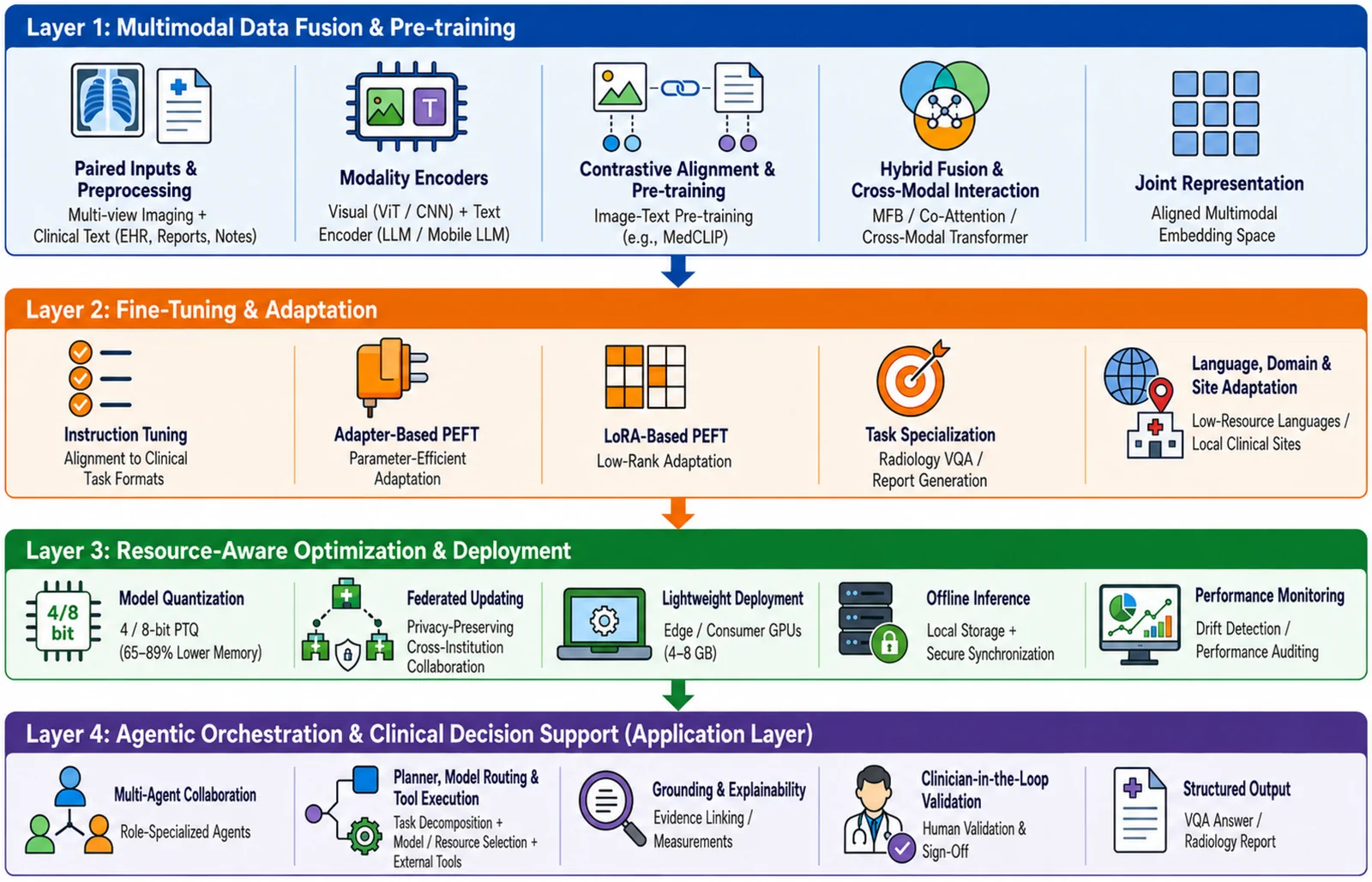
Proposed four-layer conceptual framework for multimodal medical diagnosis in low-resource healthcare settings, structured according to the contemporary vision–language model development and application pipeline. Layer 1 integrates multimodal data preparation, modality-specific encoding, hybrid fusion, cross-modal alignment, and joint pre-training. Layer 2 introduces a dedicated fine-tuning and adaptation stage comprising instruction tuning, task specialization, adapters, parameter-efficient fine-tuning (PEFT), LoRA, and language-, domain-, and site-specific adaptation. Layer 3 covers resource-aware optimization and deployment, including quantization, efficient inference, offline operation, privacy-preserving implementation, and post-deployment monitoring. Layer 4 positions agentic orchestration at the final application layer, where the system routes cases among adapted models, specialist agents, external tools, and clinical resources to provide clinician-supervised decision support. The framework is conceptual and has not been empirically validated.

We propose a conceptual framework (Figure 1) that follows the contemporary development-to-application sequence of a medical vision–language model. Layer 1 integrates multimodal data preparation, modality-specific encoding, hybrid fusion, cross-modal alignment, and joint pre-training. Layer 2 provides a dedicated stage for fine-tuning and adaptation, including instruction tuning, task specialization, adapters, PEFT, LoRA, and language-, domain-, and site-specific adaptation. Layer 3 applies resource-aware optimization and establishes the operational deployment environment. Layer 4 is the final application layer, in which agentic orchestration selects and coordinates adapted models, specialist agents, external tools, and clinical resources to produce clinician-supervised decision support. The framework is conceptual rather than empirically validated and is intended to organize future deployment-oriented research, not to prescribe an autonomous clinical system.

### 5.1 Layer 1: multimodal data fusion and pre-training

The first layer establishes the joint representation on which all later stages depend. It begins with acquisition, de-identification, quality control, preprocessing, and harmonization of heterogeneous radiological images and clinical text. Modality-specific encoders then transform images and language into compatible representations. Within this same layer, early or hybrid fusion modules, cross-modal attention, contrastive alignment, and joint pre-training learn relationships between image regions and clinical concepts. These operations are grouped together because fusion and alignment are not a post-training application stage; they are central mechanisms through which the VLM learns its shared multimodal representation. Multi-view imaging provides complementary diagnostic signals (13), while contrastive pre-training can reduce dependence on task-specific labels (16). Data-quality controls are essential in low-resource settings, where images may be low resolution, reports may be incomplete, and equipment and acquisition protocols may vary across sites.

### 5.2 Layer 2: fine-tuning and adaptation

The second layer specializes the pretrained model for clinical tasks, populations, languages, and institutions. It includes supervised task-specific fine-tuning for VQA, report generation, classification, or grounding; instruction tuning that aligns model outputs with clinical task formats (17, 39); and parameter-efficient adaptation through adapters, PEFT, and LoRA (32, 33). Language-, domain-, and site-specific adaptation are also located in this layer because they modify the model’s learned behavior before deployment. This dedicated stage separates adaptation from deployment optimization: PEFT and LoRA reduce the number of trainable parameters and make specialization feasible, but they are training and adaptation methods rather than inference-time deployment mechanisms.

### 5.3 Layer 3: resource-aware optimization and deployment

The third layer converts an adapted model into an operational system that fits the target clinic’s hardware, connectivity, privacy, and maintenance constraints. Post-training quantization can reduce dynamic inference memory to a sub-8 GB profile (34), while pruning, distillation, caching, and hardware-aware runtime selection can further reduce latency and energy demand where validated implementations are available. Deployment design also includes local or edge inference, offline operation, secure storage and delayed synchronization, access control, audit logging, and fallback behavior during connectivity or power interruption. Where institutions continue collaborative model improvement after deployment, privacy-preserving federated updating and aggregation are operationalized here without centralizing patient data (30, 31). Monitoring for calibration drift, performance degradation, data shift, and hardware failure is part of this layer because these risks emerge after the adapted model is placed in its operational environment. PEFT and LoRA are intentionally excluded from this layer and retained in Layer 2.

### 5.4 Layer 4: agentic orchestration and clinical decision support

The fourth and highest layer is the application layer. It does not create the base representation or fine-tune the model; instead, it selects and coordinates the trained, adapted, and resource-optimized components needed for a particular case. An orchestrator may route inputs among a radiology VLM, specialist agents, retrieval systems, patient-history services, calculators, or other validated clinical tools; request additional evidence when confidence is insufficient; reconcile outputs; and present a traceable recommendation. Role-specialized collaboration (23) and grounding or memory mechanisms (20) can support decomposition and traceability, but they also introduce coordination error and tool-dependency risks. For this reason, the output is framed as *clinical decision support*, not an autonomous clinical decision. Clinician oversight, uncertainty communication, escalation rules, and an audit trail form the final safety boundary of the framework.

## 6 Conclusion

This narrative review did not apply formal systematic review protocols; search and selection procedures are described in Section 1. Selection bias toward high-profile English-language papers and positive results is likely. Real-world deployment evidence from LMIC settings is sparse in the reviewed literature, which limits how broadly these findings can be applied.

Multimodal medical AI for radiology has progressed substantially since 2018, from shallow attention-based fusion to cross-modal transformers, memory-augmented report generation, and instruction-tuned multi-agent systems. On benchmark tasks, these models achieve high accuracy and competitive BLEU and ROUGE scores. However, benchmark performance and clinical validity in low-resource settings are not the same thing. No reviewed model has been prospectively validated in an LMIC clinic, and the hardware requirements of the best-performing models A100 GPUs, closed cloud APIs, multi-GPU training clusters are incompatible with district hospital infrastructure.

The most concrete progress toward low-resource deployment comes from three directions: post-training quantization reducing inference memory to 1.68–5.18 GB on consumer hardware (34); parameter-efficient fine-tuning via adapters and LoRA (32, 33) enabling domain adaptation without full retraining; and federated learning (30, 31) supporting cross-institution model improvement without data centralization. Multilingual coverage, calibration on local patient populations, and prospective clinical validation remain the most significant open problems.

Bridging the gap between benchmark performance and clinical utility in low-resource settings requires targeting specific, measurable deployment criteria: sub-8 GB inference memory, fully offline operation, language coverage beyond English, and validation against local radiologist reference diagnoses. Progress on these criteria, rather than further scaling, is what will determine whether multimodal medical AI reaches the settings where it is most needed.
