# Supplementary material for: Multimodal medical diagnosis: a mini review of LLM–vision fusion models in low-resource healthcare settings
Source: Front Digit Health. 2026 Sep 2;8:1862236. doi: 10.3389/fdgth.2026.1862236 (PMC13579002; doi:10.3389/fdgth.2026.1862236)
Supplement: Supplementary file 1 [file Table1.pdf]

Supplementary Table S1

Verified contextual and methodological references used outside the tiered (Tier 1–3) comparative evidence set ($n = 11$).

No.	Study	Title	DOI / Link	Use in this review and formal status
1	Malpani & Moodley, 2025	Large Multimodal Models: Boon or Burden for Low- and Middle-Income Countries	https://doi.org/10.1093/inthealth/ihae073	Used to frame the gap between strong benchmark results and practical deployment in LMIC settings (Introduction, §2.2, and §4.5). This is a contextual source and is not included in the Tier 1–3 evidence set.
2	Ben Abacha & Demner-Fushman, 2019	VQA-Med: Overview of the Medical Visual Question Answering Task at ImageCLEF 2019	https://ceur-ws.org/Vol-2380/paper_272.pdf	Cited to document an early medical-VQA benchmark and the provenance of image-question-answer tasks. It provides methodological background and is not evaluated as a Tier 1–3 study.
3	Zhang et al., 2022	Contrastive Learning of Medical Visual Representations from Paired Images and Text	https://proceedings.mlr.press/v182/zhang22a.html	Provides methodological background on paired image–text contrastive learning that preceded MedCLIP. It is cited for architectural context and is not included in the Tier 1–3 evidence set.
4	Singhal et al., 2023	Large Language Models Encode Clinical Knowledge	https://doi.org/10.1038/s41586-023-06291-2	Provides background on instruction-tuned clinical LLMs that informed later multimodal systems such as Med-PaLM M. It is a contextual source outside the Tier 1–3 evidence set.
5	Rajpurkar et al., 2017	CheXNet: Radiologist-Level Pneumonia Detection on Chest X-Rays with Deep Learning	https://doi.org/10.48550/arXiv.1711.05225	Serves as a historical unimodal chest X-ray baseline in the Introduction. Because it is not a multimodal study, it falls outside the Tier 1–3 evidence set.
6	Lee et al., 2020	BioBERT: A Pre-trained Biomedical Language Representation Model for Biomedical Text Mining	https://doi.org/10.1093/bioinformatics/btz682	Cited as a foundational biomedical-language model used to explain the clinical-NLP lineage. It provides background only and is outside the Tier 1–3 evidence set.
7	Alsentzer et al., 2019	Publicly Available Clinical BERT Embeddings	https://doi.org/10.18653/v1/W19-1909	Cited as a clinical text-encoder baseline that helps establish the development path toward multimodal medical models. It is contextual and is not part of the Tier 1–3 evidence set.
8	Seyyed-Kalantari et al., 2021	Underdiagnosis Bias of Artificial Intelligence Algorithms Applied to Chest Radiographs in Under-Served Patient Populations	https://doi.org/10.1038/s41591-021-01595-0	Supports the discussion of underdiagnosis bias and domain shift in §4.2. It provides methodological evidence but is not one of the studies compared in the Tier 1–3 evidence set.
9	Liu et al., 2023	Visual Instruction Tuning (LLaVA)	https://doi.org/10.52202/075280-1516	Identifies the parent architecture of LLaVA-Med, which is included as a Tier 2 comparator. The original LLaVA paper is cited for architectural provenance and is not itself part of the Tier 1–3 evidence set.
10	Hu et al., 2022	LoRA: Low-Rank Adaptation of Large Language Models	https://openreview.net/forum?id=nZeVKeeFYf9	Defines LoRA, the parameter-efficient adaptation method discussed in the review. It supplies a methodological foundation and is not an included Tier 1–3 study.
11	Rieke et al., 2020	The Future of Digital Health with Federated Learning	https://doi.org/10.1038/s41746-020-00323-1	Provides the general federated-learning framework underlying the Tier 1 federated radiology study. It is methodological background and is not independently included in the Tier 1–3 evidence set.
